# Supplementary material for: A Possible Modulator of Vitiligo Metabolic Impairment: Rethinking a PPARγ Agonist
Source: Cells. 2022 Nov 12;11(22):3583. doi: 10.3390/cells11223583 (PMC9688513; doi:10.3390/cells11223583)
Supplement: Supplementary file 1 [file cells-11-03583-s001.zip › cells-1986274-supplementary.pdf]

**Supplementary Table S1. Primers for Real Time Quantitative Polymerase Chain Reaction**

| Target Gene | Foward Primer | Reverse primer |
|---|---|---|
| βactin | 5’-GACAGGATGCAGAAGGAGATTACT-3’ | 5’-TGATCCACATCTGCTGGAAGGT-3’ |
| ACADM | 5’-TGGTGAATATCCAGTCCCCCT-3’ | 5’-CCAAGTCCAAGACCTCCACA -3’ |
| ACADS | 5’-CCTCAGCGAACCAGGGAAC-3’ | 5’-TTCAGAACCCATGAGTCGCC-3’ |
| ACOX | 5’-CGCCGAGAGATCGAGAACAT-3’ | 5’-GACAGCCACCTCATAACGCT-3’ |
| Catalase | 5’-GGGCAACAGGCAGATTTG-3’ | 5’-CTGCTCCTTCCAGTGCTG-3’ |
| CD36 | 5’-CGGCTGCAGGTCAACCTATT -3’ | 5’-GGTCCCAGTCTCATTAAGCCA-3’ |
| Cox2 | 5’-GAATCATTCACCAGGCAAATTC-3’ | 5’-CTGTACTGCGGGTGGAACATT-3’ |
| CXCR3B | 5’-TGCCAGGCCTTTACACAGC -3’ | 5’-TCGGCGTCATTTAGCACTTG-3’ |
| Glut1 | 5’-TGGCATCAACGCTGTCTTCT-3’ | 5’-AGCCAATGGTGGCATACACA3’ |
| Glut3 | 5’-CGAACTTCCTAGTCGGATTG-3’ | 5’-AGGAGGCACGACTTAGACAT-3’ |
| Glut4 | 5’-TCAGAGACTCCAGGATCGGTT -3’ | 5’-GGTTCCCCATCTTCGGAGC-3’ |
| Hexo2 | 5’-CCTCGGTTTCCCAACTCTGC -3’ | 5’-GGTCAACCTTCTGCACTTGG-3’ |
| HMGB1 | 5’-GTGCCTCGCTGAGGAAAAAT-3’ | 5’-TCCTCCCGACAAGTTTGCAC-3’ |
| HO-1 | 5’-CAGTGCCACCAAGTTCAAG-3’ | 5’-CAAGACTGCGTTCCTGCT-3’ |
| IGFBP3 | 5’-CACTCTGGGAACCTATAAAG-3’ | 5’-ATTACTTGTGATGCCTCTG -3’ |
| IGFBP5 | 5’-CGGGGTTTGCCTCAACGAA-3’ | 5’-TCTTGGGGGAGTAGGTCTCCT-3’ |
| IGFBP7 | 5’-GCTGTGAGGTCATCGGAATC-3’ | 5’-ACCAGGCAGGAGTTCTGTC-3’ |
| MICA | 5’-GTCCTGAGGAGAACAGTGCC -3’ | 5’-GGGATAGAAGCCAGAAGCCC-3’ |
| MICB | 5’-GAGAAGGTGGCGACGTAGG-3’ | 5- GACTGTGGGGCTCAGCG -3’ |
| MITF | 5’-ATGGACGACACCCTTTCTC-3’ | 5’-GGAGGATTCGCTAACAAGTG-3’ |
| OGDH | 5’-CCCTGGGGATTTTGGATGCT-3’ | 5’-CCAGGCCATAGAACCCAAGT-3’ |
| PC | 5’-TTGCCTCCGCAGATAGTGTC-3’ | 5’-GGATTCCCAGGAGCCTCAG-3’ |
| PD-L1 | 5’-TGCAGGGCATTCCAGAAAGA-3’ | 5’-ACCGTGACAGTAAATGCGTTC-3’ |
| PDK4 | 5’-CCCTGAGAATTATTGACCGCCT-3’ | 5’-CCGTAACCAAAACCAGCCAAA-3’ |
| PGC1α | 5’-TCATTCAGGAGCTGGATGGC-3’ | 5’’GAGCAGCACACTCGATGTCA-3’J |
| PKM1,2 | 5’-TCAGCGCCGGAGGACC-3’ | 5’-TGCTCCAGGAATGTGTCAGC-3’ |
| p16 | 5’-GAGCAGCATGGAGCCTTC-3’ | 5’-CATCATCATGACCTGGAT-3’ |
| p21 | 5’-CGCTCTTACATCTTCTGCCTTAGTC-3’ | 5’-AACCTCTCATTCAACCGCCCTAG-3’ |
| SOD2 | 5’-GGCTGCATCTGTTGGTGTCC-3’ | 5’-TCCCACACATCAATCCCCAG-3’ |
| UCP2 | 5’-AGTCCGGTTACAGATCCAAGG -3’ | 5’-TCAGAATGGTGCCCATCACA-3’ |
| VDAC | 5’-CCCGGAAGGCAGAAGATGG-3’ | 5’-TTGGTGAAGACATCCCTGGC-3’ |

**Supplementary Table S2. Primary Antibodies**

| Antibody | Diluition | |
|---|---|---|
| βactin | 1:10000 | SIGMA-Aldrich |
| AMPK | 1:1000 | CellSignaling |
| p-AMPK | 1:1000 | CellSignaling |
| b- FGF | 1:1000 | Upstate Biotechnology |
| CD36 | 1:1000 | CellSignaling |
| Erk | 1:1000 | SantaCruz |
| p- Erk | 1:1000 | CellSignaling |
| Fibronectin | 1:500 | SantaCruz |
| Hexo2 | 1:1000 | CellSignaling |
| HGF | 1:1000 | CellSignaling |
| Hsp70 | 1:1000 | CellSignaling |
| IGFBP3 | 1:1000 | CellSignaling |
| MITF | 1:1000 | CellSignaling |
| m TOR | 1:1000 | CellSignaling |
| p m TOR | 1:1000 | CellSignaling |
| Total OXPHOS | 1:1000 | Abcam |
| PDK4 | 1:1000 | CellSignaling |
| PKM1,2 | 1:1000 | CellSignaling |
| UCP2 | 1:1000 | CellSignaling |
